# *Bifidobacterium animalis* subsp. *Lactis* BL‐99 Improves Maternal and Fetal Immune Responses and Pregnancy Outcomes in Pregnant Antibiotics‐Treated Mice

**DOI:** 10.1002/mnfr.70220

**Published:** 2025-09-04

**Authors:** Marijke M. Faas, Lieske Wekema, Carolien A. van Loo‐Bouwman, Gisela A. Weiss, Wei‐lian Hung, Bart J. de Haan, Alexandra M. Smink

**Affiliations:** ^1^ Department of Pathology and Medical Biology University of Groningen and University Medical Center Groningen Groningen the Netherlands; ^2^ Department of Obstetrics and Gynaecology University of Groningen and University Medical Center Groningen Groningen the Netherlands; ^3^ Yili Innovation Center Europe Wageningen the Netherlands; ^4^ National Center of Technology Innovation For Dairy Hohhot China

**Keywords:** antibiotics, immune response, pregnancy, probiotics

## Abstract

The maternal gut microbiome is involved in adapting immune responses to the presence of the semiallogeneic foetus. We have previously shown that antibiotics‐induced gut dysbiosis, alterations in the maternal immune response and decreased foetal and placental weight. Here, we tested whether Bifidobacterium animalis subsp. *lactis* BL‐99 (BL‐99) could improve antibiotics‐induced gut dysbiosis, maternal immune responses and foetal and placental weight. To do so, pregnant mice received antibiotics in their drinking water (day 9‐16) and BL‐99 via oral gavage (day 9‐18). After sacrifice (day 18) immune responses were measured using flowcytometry. BL‐99 increased placental weight in antibiotics‐treated pregnant mice. BL‐99 did not significantly change the maternal microbiome, but improved maternal immune responses by decreasing splenic Th1 cells and Treg cells, and increasing FoxP3/RoRγT double‐positive cells in the Peyer's patches to levels observed in control pregnant mice. BL‐99 also improved monocyte subsets and activation status. Additionally, BL‐99 changed foetal monocyte subsets and activational status and increased foetal splenic Th cells. We thus showed that the effect of antibiotics treatment on immune cells and placental weight was mitigated by supplementation of BL‐99. We suggest that pregnancy complications associated with a disturbed microbiome and immune responses, such as preeclampsia or obese pregnancies, could benefit from BL‐99 supplementation. This should be tested in future studies.

## Graphical abstract

Pregnant mice treated with antibiotics showed gut dysbiosis, which is associated with reduced fetal and placental weight, and disrupted maternal and fetal immune responses. Supplementation via oral gavage with *Bifidobacterium animalis* subsp. *lactis* BL‐99 increased *B. animalis* levels in the gut, but did not fully restore microbiome balance. However, BL‐99 supplementation significantly improved placental weight and immune responses, both in the mother and fetus, without affecting fetal weight.

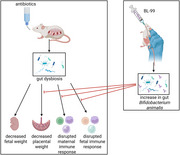

**Abbreviations**
AB: antibiotic
BL‐99: *Bifidobacterium animalis* subsp. lactis BL‐99
cfu: colony forming units
dFCS: decomplemented fetal calf serum
NK: natural killer
OTU: operational taxonomic unit
PP: Peyer's patches
Th: T helper
ThWA: three‐way ANOVA
TWA: two‐way ANOVA

## 1 Introduction

Pregnancy is a unique immunological challenge in which the maternal immune response adapts to tolerate the developing semiallogeneic fetus. In the peripheral circulation during both human and mouse pregnancy, the immune response is shifted away from a T helper (Th)1 type immune response to a Th2 type immune response, while regulatory T cells are increased and Th17 cells are decreased [1, 2, 3]. Moreover, also changes in the innate immune response are observed, characterized by increased numbers of monocytes, as well as increased activation of these cells and changes in monocyte subsets [4, 5, 6]. At the maternal‐fetal interface, specific immune cells, such as uterine natural killer (NK) cells and macrophages, are present to help in placental development and fetal tolerance [7]. The immunological adaptations are important for a healthy pregnancy, since maladaptation of the immune response to pregnancy is associated with pregnancy complications as preeclampsia, fetal growth retardation, and recurrent pregnancy loss [2, 8, 9].

The mechanisms inducing the adaptations of the maternal immune response are complex, with involvement of maternal hormones, such as progesterone [10] and the placenta, which secretes various factors into the maternal circulation, such as cytokines, exosomes, and DNA, that can affect the maternal immune response [11, 12, 13]. Recently, we have shown that the maternal gut microbiome is also important, since adaptations in the maternal immune response are different in germ‐free mice versus conventional mice [14]. Moreover, disturbances of the maternal gut microbiome by antibiotics (ABs) treatment or obesity resulted in aberrant adaptations of the maternal immune response in pregnant mice, which is associated with adverse pregnancy outcomes, such as decreased placental and fetal weight [15, 16].

In the present paper, we hypothesize that supplementation with probiotics in pregnancies with a disturbed microbiome may improve the microbiome and therefore improve maternal immune responses and pregnancy outcome. Thus, AB‐treated pregnant mice were supplemented with the probiotic *Bifidobacterium animalis* subsp. *lactis* BL‐99 (BL‐99, CGMCC No. 15650) during and after AB treatment. This probiotic *Bifidobacterium* is known to have several health effects [17, 18, 19]. By studying the effects on the maternal microbiome and the maternal immune response, we provide some mechanistic insight into the effects of AB treatment and BL‐99 supplementation on pregnancy outcome. As the maternal microbiome may also affect fetal immune responses, we also evaluated the effect of AB treatment and BL‐99 supplementation on fetal immune responses.

## 2 Experimental Section

### 2.1 Experimental Setup

Conventional female C57BL/6OlaHsd mice (2.5 months old) were ordered from Envigo (Horst, the Netherlands), between January 2021 and August 2022. In view of the reduction of use of animals, data of part of the control and AB‐treated pregnant and nonpregnant mice, that is, mice without supplementation with BL‐99, were used from our previous paper [16]. Mice were placed on a standardized diet (D10012Mmi; Research Diets Inc., New Brunswick, USA) for 3 weeks before starting experiments. They remained on the diet throughout the experiments. Mice were housed together (3–5 mice per cage) in individually ventilated cages, with a light regime of 12 h light and 12 h dark with free access to food and water. Mice were handled in flow cabinets. Vaginal smears were taken to follow the estrus cycle, and on the day of pro‐estrus the mice were allowed to mate (male C57BL/6OlaHsd mice, 3–6 months old; Envigo) overnight. The next morning was Day 0 of pregnancy. To confirm pregnancy, mice were weighed on Day 8 of pregnancy, and a weight gain of more than 1.5 g indicated pregnancy. Mice were mated in groups of 3–5 mice at a time, and upon confirmation of pregnancy, randomly assigned to one of the four pregnant groups. Nonpregnant mice were used as controls and randomly assigned to one of the four nonpregnant groups. In view of the different groups and different treatments, it was not possible to blind the investigators during the experiments. All experiments were approved by the Central Committee of Animal Experimentation in the Netherlands (AVD1050020198488) and performed according to their guidelines.

Pregnant mice were treated with AB in their drinking water from Day 9 until Day 16 of pregnancy. In addition, they were supplemented with or without BL‐99 (kindly provided by Yili Innovation Center Europe, Wageningen, the Netherlands) from Day 9 until Day 18 via oral gavage. The nonpregnant control mice received AB for 7 days and were given BL‐99 from the start of AB treatment until 2 days after stopping AB treatment. Together with the nontreated control groups, this resulted in 8 experimental groups of mice, which all contained 8–9 biological replicates: pregnant AB‐treated mice: *n* = 8 (4 of these mice were randomly taken from our previous paper [16] and 4 mice are new); pregnant AB/BL‐99 treated mice: *n* = 8 (these mice are new, i.e., they have not been used in a previous study); pregnant control mice: *n* = 8 (4 of these mice were randomly taken from our previous paper [16] and 4 mice are new); pregnant control mice supplemented with BL‐99: *n* = 8 (these mice are new); nonpregnant AB‐treated mice: *n* = 8 (3 of these mice were randomly taken from our previous paper [16] and 5 mice are new); nonpregnant AB/BL‐99 treated mice: *n* = 9 (these mice are new); nonpregnant control mice: *n* = 8 (these mice are new); nonpregnant control mice supplemented with BL‐99: *n* = 9 (these mice are new) (see Figure S1).

Two days before the start of the AB/BL‐99 treatment (Day −2), 5 days after the start of the AB/BL99 treatment (Day 5), and 2 days after stopping AB treatment (Day +2), fecal pellets were collected. For this, mice were placed in an empty cage for a few minutes until defecation; all pellets collected were immediately snap‐frozen in liquid nitrogen and stored at −80°C. From nonpregnant control mice that were not treated with AB/BL‐99, we only collected fecal pellets at Day −2. At Day +2 (which is Day 18 of pregnancy for pregnant mice), the mice were sacrificed by heart puncture under isoflurane anesthesia. Blood from the heart was collected in EDTA tubes (BD Biosciences, Franklin Lakes, USA). Spleens and Peyer's patches (PP) were collected in RMPI (Thermo Fisher Scientific, USA), supplemented with 10% decomplemented fetal calf serum (dFCS; Serana, Pessin, Germany) until cell isolation within 2 h). After counting the number of fetuses and the number of live fetuses and weighing individual fetuses and placentas, we collected fetal blood in EDTA capillaries (Greiner Bio‐One; Alphen aan den Rijn, the Netherlands) for analyzing fetal monocytes. We also collected the fetal spleen (in RMPI supplemented with 10% dFCS) for analyzing fetal T cells. Since we can only collect limited amounts of blood and cells from the fetal spleen, the blood and spleens from all fetuses of one mother were pooled.

### 2.2 AB Treatment

ABs were given in the drinking water according to our previous paper [16, 20]. The AB consisted of 5 mg/mL bacitracin (Merck, Darmstadt, Germany), 1.25 µg/mL primaricin (a stock of 5 mg/mL in acetic acid was used; Merck), and 5 mg/mL neomycin (Merck). Control mice received regular drinking water.

### 2.3 Dosage Information

BL‐99 was supplemented via daily oral gavage. All handling with BL‐99 during culturing was done in an anaerobic chamber (Don Whitley Scientific Limited, Bingley, UK). BL‐99 was freshly cultured every day, anaerobically, overnight, and under continuous shaking at 37°C, after which an optical density between 0.74 and 1 was reached. This corresponded to a concentration between 1 × 10^8^ colony forming units (cfu) and 20 × 10^8^ cfu BL‐99/mL. The culture was centrifuged, and the pellet was taken up in sterile PBS to reach a concentration of 1 × 10^8^ cfu BL‐99 per 150 µl. Each mouse was given 150 µL of BL‐99 daily. Controls were not treated with BL‐99. This dose was based on pilot experiments and a previous paper using BL‐99 in nonpregnant mice [19]. The human equivalent would be about 2 × 10^11^ cfu per day.

### 2.4 Microbiota Measurement

The fecal DNA isolation and microbiota measurements were done by Baseclear (Leiden, the Netherlands) as described previously [16]. For the operational taxonomic unit (OTU) classification, paired‐end sequence reads were collapsed into so‐called pseudo reads using sequence overlap with USEARCH version 9.2. Classification of these pseudo reads was performed based on the results of alignment with SNAP version 1.0.23 against the RDP database version 11.5 for bacterial organisms [21, 22, 23].

### 2.5 Universal Bacterial Quantitative Polymerase Reaction

The fecal DNA was also used for measuring total bacteria levels by qPCR. To this end, the universal bacteria primers, forward primer 5′‐TCCTACGGGAGGCAGCAGT‐3′ and reverse primer 5′‐GGACTACCAGGGTATCTAATCCTGTT‐3′, were used as previously described [16]. Only samples with Ct values <35 were considered positive for amplification. The Ct was measured, and the 2^−Ct^ was used as a measure for bacterial counts.

### 2.6 Isolation and Staining of T Cells and Dendritic Cells/Macrophages From the Spleens and PP

After sacrifice, spleens (fetal and maternal) and maternal PP were taken from each mouse, and cells were isolated from these tissues as previously described [16]; red blood cells were lysed if necessary [16]. After isolation, cells were counted, and about 1 × 10^6^ cells were used for staining. Samples were stained in 96 well plates. The intra‐ and extracellular antibodies used for staining of T cell subsets are shown in Table S1. Extracellular antibodies were diluted in 25 µL FACS buffer (Dulbecco's Phosphate Buffered Saline with 2% dFCS). The intracellular antibody panel was diluted in 50 µL FACS permeabilization solution (eBioscience, Vienna, Austria). The staining protocol used was described previously [16].

### 2.7 Staining of Blood Monocytes

Maternal and fetal EDTA blood was used for staining of monocytes. We stained for monocyte subsets (classical, nonclassical, and intermediate monocytes) as well as for their activation status (using antibodies against CD80 and MHCII). The antibodies used are shown in Table S1. All antibodies were diluted in 25 µL FACS buffer supplemented with 37.2 mg EDTA (Merck) per 100 mL. For the staining, 200 µL of blood was used, which was diluted with 200 µL of RPMI with 10% dFCS. Staining was done according to the protocol described previously [16].

### 2.8 Flow Cytometry

All our samples were analyzed using the FACSverse flow cytometer system (BD Biosciences, Franklin Lakes, USA) with FACSverse software. We used FlowJo software (version 10; FlowJo, LLC, Oregon, USA) with the gating strategy as described previously [16].

### 2.9 Statistics

During the flow cytometry and microbiome analysis, investigators were blinded to the outcome as much as possible, since mice were numbered consecutively and not according to treatment. Graphpad Prism version 10.2.2 for Windows (GraphPad Software, Boston, MA, USA, “www.graphpad.com”) was used for statistical testing. For evaluating the Simpson diversity index and for doing PCA, and Permanova we used the program Past4 [24]. For Permanova, we used Bray Curtis distance and for pairwise comparisons, sequential Bonferroni correction was applied.

For Simpson diversity index, BL‐99 abundance, maternal, fetal, and placental weight as well as for immunological data, data were first tested for normality using Kolmogorov–Smirnov test. If data were not normally distributed, they were log‐transformed before performing the statistical testing.

For evaluating the effect of AB and BL‐99 on fetal and placental weight in pregnant mice and fetal immune cells populations, we used the two‐way ANOVA (TWA) followed by Sidak's multiple comparison's test, which corrects for multiple testing.

For evaluating the differences in Simpson diversity index, total bacterial number, and BL‐99 abundance between the groups, we used three‐way ANOVA (ThWA) to evaluate the effect of AB treatment, BL‐99 supplementation, and day of treatment. This was followed by Sidak's multiple comparison's test to evaluate differences between Day −2 and Days 5 and +2 within the groups.

For evaluating maternal immune cell populations in the spleen and PP, we used ThWA to evaluate the effects of pregnancy, AB‐treatment, and BL‐99 supplementation. Using a post‐test (the Sidak's multiple comparison's test), we tested significant differences between control and AB‐treated mice with or without BL‐99 supplementation (to evaluate the effect of AB‐treatment, with or without BL‐99 supplementation). To evaluate the effect of BL‐99 supplementation per se, we tested whether the difference between control and BL‐99 supplemented mice was significant.

Spearman's correlation was used to evaluate the correlation between the abundance of *B. animalis* species and immune cell subsets/activation markers. Correlations were evaluated in pregnant AB‐treated mice with or without BL‐99 supplementation, pregnant control mice with or without BL‐99 supplementation, nonpregnant AB‐treated mice with or without BL‐99 supplementation, and control nonpregnant mice with or without BL‐99 supplementation. Data were considered significantly different if *p* < 0.05.

## 3 Results

### 3.1 The Effect of AB and BL‐99 on Maternal, Fetal, and Placental Weight

AB‐treatment decreased maternal weight on Days 1, 2, 3, 4, and 5 after the start of the treatment as compared with control untreated pregnant mice (Figure S2). BL‐99 supplementation did not affect maternal weight in the AB group nor in the control group. In nonpregnant mice, AB‐treatment decreased the weight of the mice during AB‐treatment as compared with control nonpregnant mice. Supplementation with BL‐99 affected the weight of the AB‐treated mice: the first 3 days after treatment weight was increased as compared with AB‐treated mice, while from Day 6 of treatment, weight was significantly decreased as compared to AB‐treated mice.

**FIGURE 1:**
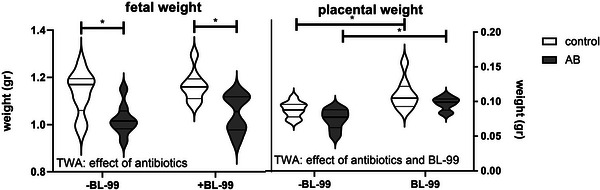
The effect of treatment with antibiotics (AB) and *Bifidobacterium animalis* subsp. *lactis* BL‐99 (BL‐99) on the fetal (left) and placental weight (right). Violin plots are shown for the mean fetal and placental weight of each mother. Each group contains 8 biological replicates. *Two‐way ANOVA (TWA) followed by Sidak's multiple comparisons test, *p* < 0.05.

Fetal weight was also affected by AB treatment (Figure 1). TWA analysis showed an effect of AB treatment; posttesting indicated that treatment with AB decreased fetal weight irrespective of supplementation with BL‐99. TWA also showed that both AB treatment and BL‐99 supplementation affected placental weight; AB treatment decreased placental weight, while BL‐99 supplementation increased placental weight (TWA, *p* < 0.05). Posttesting revealed that BL‐99 supplementation significantly increased placental weight in control and AB‐treated pregnant mice (Sidak's multiple comparison's test, *p* < 0.05).

### 3.2 The Effect of AB and BL‐99 on the Maternal Gut Microbiota

**FIGURE 2:**
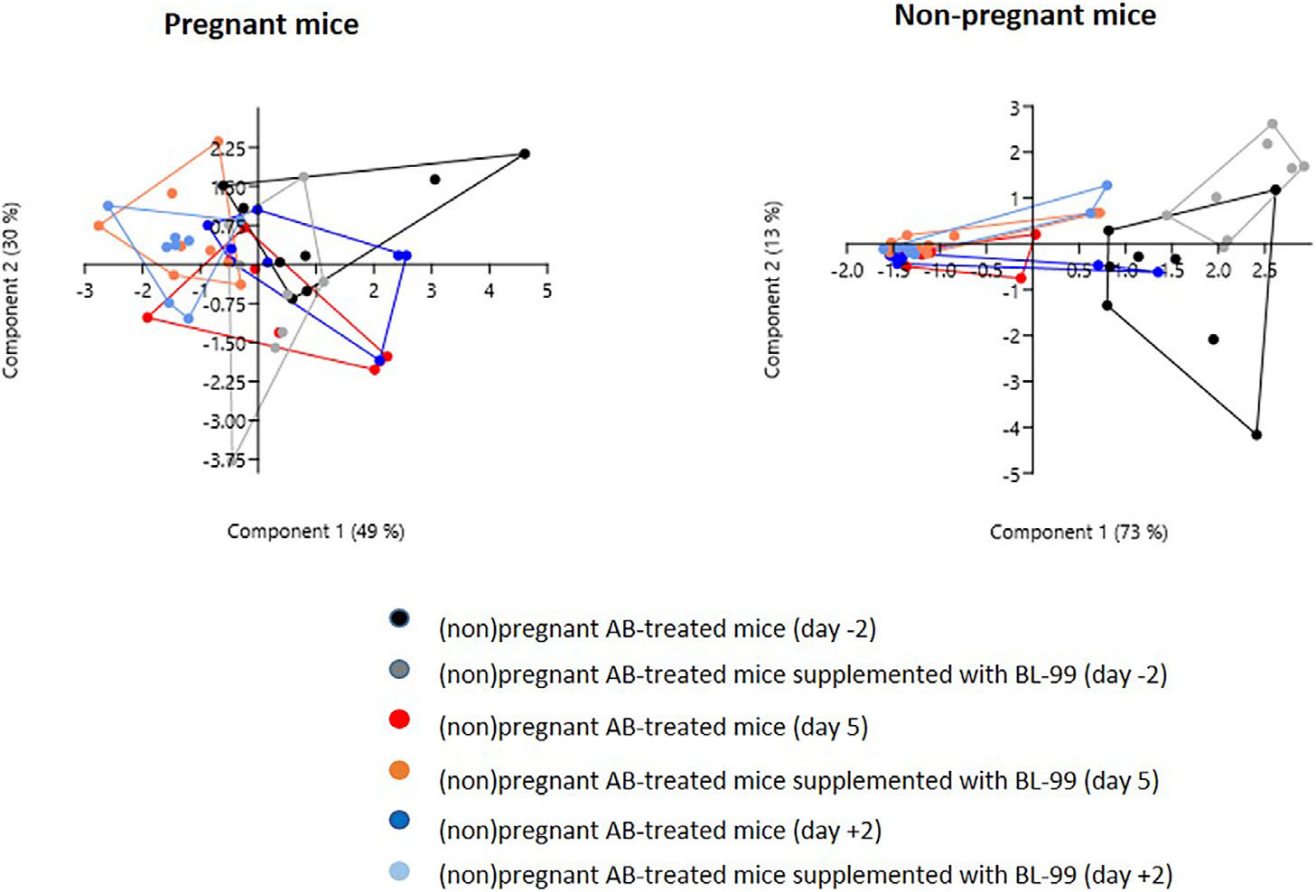
PCA plots of bacterial phyla in pregnant (left) and nonpregnant (right) mice treated with antibiotics (AB) and supplemented with or without *Bifidobacterium animalis* subsp. *lactis* BL‐99 (BL‐99). Nonpregnant AB‐treated mice, pregnant AB‐treated mice, pregnant AB/BL‐99‐treated mice: 8 biological replicates; nonpregnant AB/BL‐99‐treated mice: 9 biological replicates.

We determined the microbiota composition at phyla and genus level of all groups before the start of the treatment with AB and BL‐99 supplementation, at Day 5 during the treatment, and 2 days (Day +2) after stopping the AB‐treatment. PCA plots of the gut bacterial phyla of pregnant mice treated with AB with or without BL‐99 are shown in Figure 2 (left). There was no statistical difference in phyla composition between the groups (Permanova, *p* > 0.05). The right graph of Figure 2 shows the PCA plots of the gut bacterial phyla of nonpregnant mice treated with AB with or without BL‐99. At Day 5 and Day +2, the microbiota phyla composition was significantly different from Day −2 for both groups (BL‐99 treated and BL‐99 untreated mice; Permanova, *p* < 0.05). No differences were found on either day between BL‐99‐treated and BL‐99‐untreated mice (Permanova, *p* > 0.05). PCA plots for control pregnant and control nonpregnant mice treated with or without BL‐99 are shown in Figure S3. There were no significant differences in phyla microbiota composition between Day −2 (before treatment with BL‐99) or after treatment with BL‐99 (Days 5 and +2) (Permanova, *p* > 0.05). For pregnant control mice, there was also no difference on either day between BL‐99 treated and untreated mice (Permanova, *p* > 0.05).

**FIGURE 3:**
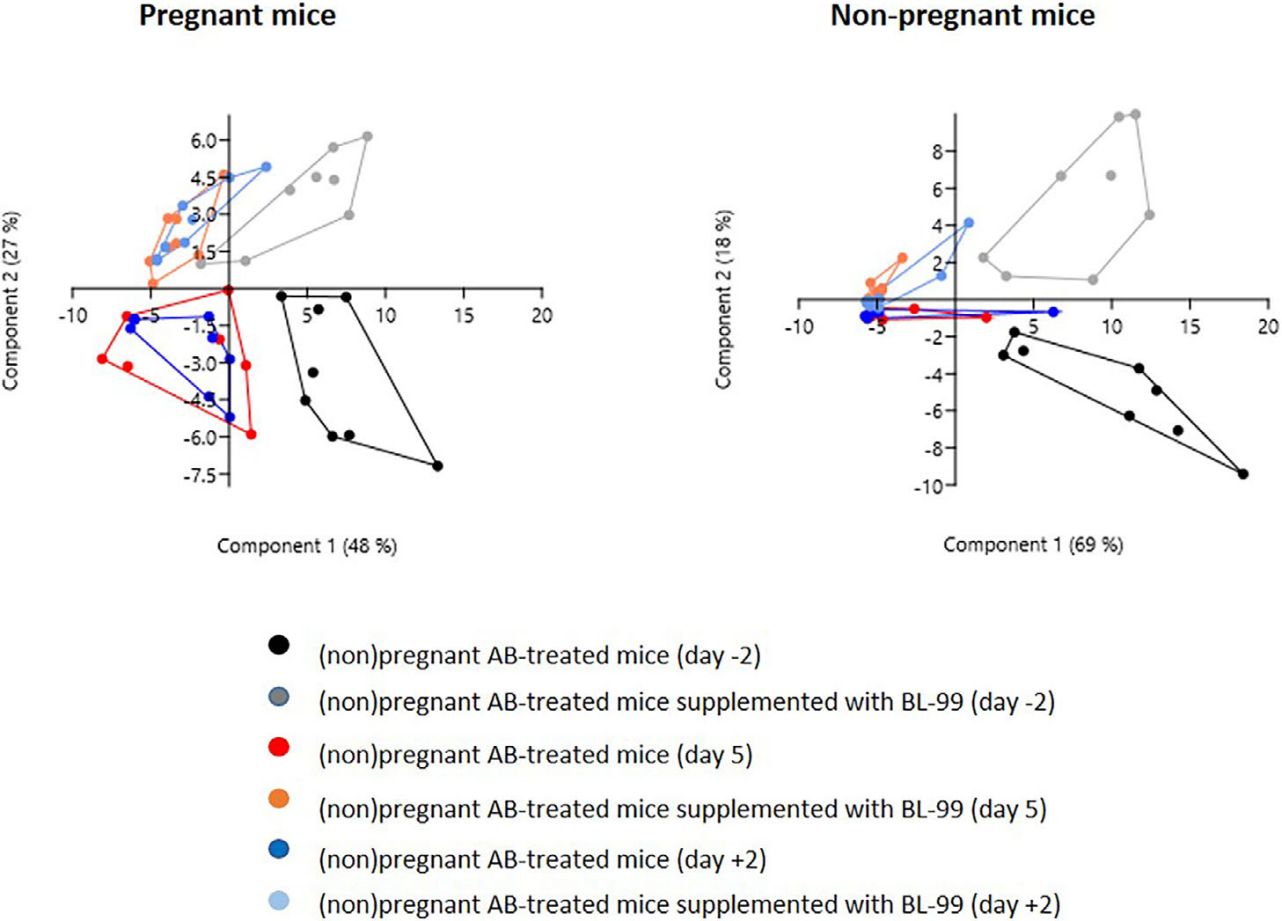
PCA plots of bacterial genera in pregnant (left) and nonpregnant (right) mice treated with antibiotics (AB) and supplemented with or without *Bifidobacterium animalis* subsp. *lactis* BL‐99 (BL‐99). Nonpregnant AB‐treated mice, pregnant AB‐treated mice, pregnant AB/BL‐99‐treated mice: 8 biological replicates; nonpregnant AB/BL‐99‐treated mice: 9 biological replicates.

Figure 3 shows the PCA plots of the bacterial genera composition of pregnant and nonpregnant mice treated with AB and with or without BL‐99. For pregnant mice (left graph), AB treatment significantly changed genera composition at Day 5, but not at Day +2 versus Day −2 (Permanova, *p* < 0.05). In AB‐treated pregnant mice supplemented with BL‐99, genera composition at Day +2, but not at Day 5, was significantly different from Day −2 (Permanova, *p* < 0.05). Permanova analysis showed no significant differences in genera composition between AB‐treated mice with or without BL‐99 treatment (*p* > 0.05). For nonpregnant mice (left graph), PCA plots showed a large effect of AB treatment in both BL‐99 supplemented and unsupplemented mice. Permanova analysis indicated that in both groups the genera composition after treatment with AB (Days 5 and +2) was significantly different from before treatment (Day −2) (Permanova, *p* < 0.05). Permanova analysis showed no significant differences in genera composition between AB‐treated nonpregnant mice with or without BL‐99 treatment (*p* > 0.05). In control pregnant and nonpregnant mice, the microbial genera were not affected by BL‐99 treatment (Permanova, *p* > 0.05) (Figure S4).

The total bacterial count was measured using qPCR, using 2^−Ct^ as a measure for bacterial count. Treatment with AB significantly decreased bacterial count in both pregnant and nonpregnant mice as compared with Day −2 in the same group (Table 1). Simultaneous supplementation with BL‐99 did not affect bacterial counts in AB‐treated pregnant or nonpregnant mice. Supplementation with BL‐99 alone also did not affect bacterial counts.

**TABLE 1:** Total bacterial count using 2^−Ct^ as a measure for bacterial count in the 8 groups of mice.

Day			Pregnant mice	Nonpregnant mice
	AB	BL‐99		
Day −2	—	—	0.074 ± 0.014	0.083 ± 0.017
	+	—	0.071 ± 0.013	0.072 ± 0.017
	—	+	0.06 ± 0.021	0.058 ± 0.013
	+	+	0.044 ± 0.012	0.086 ± 0.014
Day 5	—	—	0.079 ± 0.020	—
	+	—	0.001 ± 0.0004^a^	0.005 ± 0.0022^a^
	—	+	0.05 ± 0.013	0.057 ± 0.016
	+	+	0.0005 ± 0.0002^a^	0.0078 ± 0.003^a^
Day +2	—	—	0.049 ± 0.017	—
	+	—	0.001 ± 0.0005^a^	0.005 ± 0.0024^a^
	—	+	0.043 ± 0.028	0.040 ± 0.007
	+	+	0.0004 ± 0.0002^a^	0.0109 ± 0.004^a^

*Note*: Nonpregnant AB‐treated mice, pregnant AB‐treated mice, pregnant AB/BL‐99‐treated mice, pregnant control mice, and pregnant control mice supplemented with BL‐99 and nonpregnant control mice: 8 biological replicates; nonpregnant control mice supplemented with BL‐99 and nonpregnant AB/BL‐99‐treated mice: 9 biological replicates. For control nonpregnant mice, feces were only collected at Day −2. For pregnant mice, ThWA found an effect of antibiotics (AB)‐treatment and BL‐99 supplementation as well as an interaction between AB‐treatment and BL‐99 supplementation (*p *< 0.05).

^a^ Significantly different from Day −2 after the same treatment. Mixed effect model followed by Sidak's multiple comparisons test, *p* < 0.05).

**FIGURE 4:**
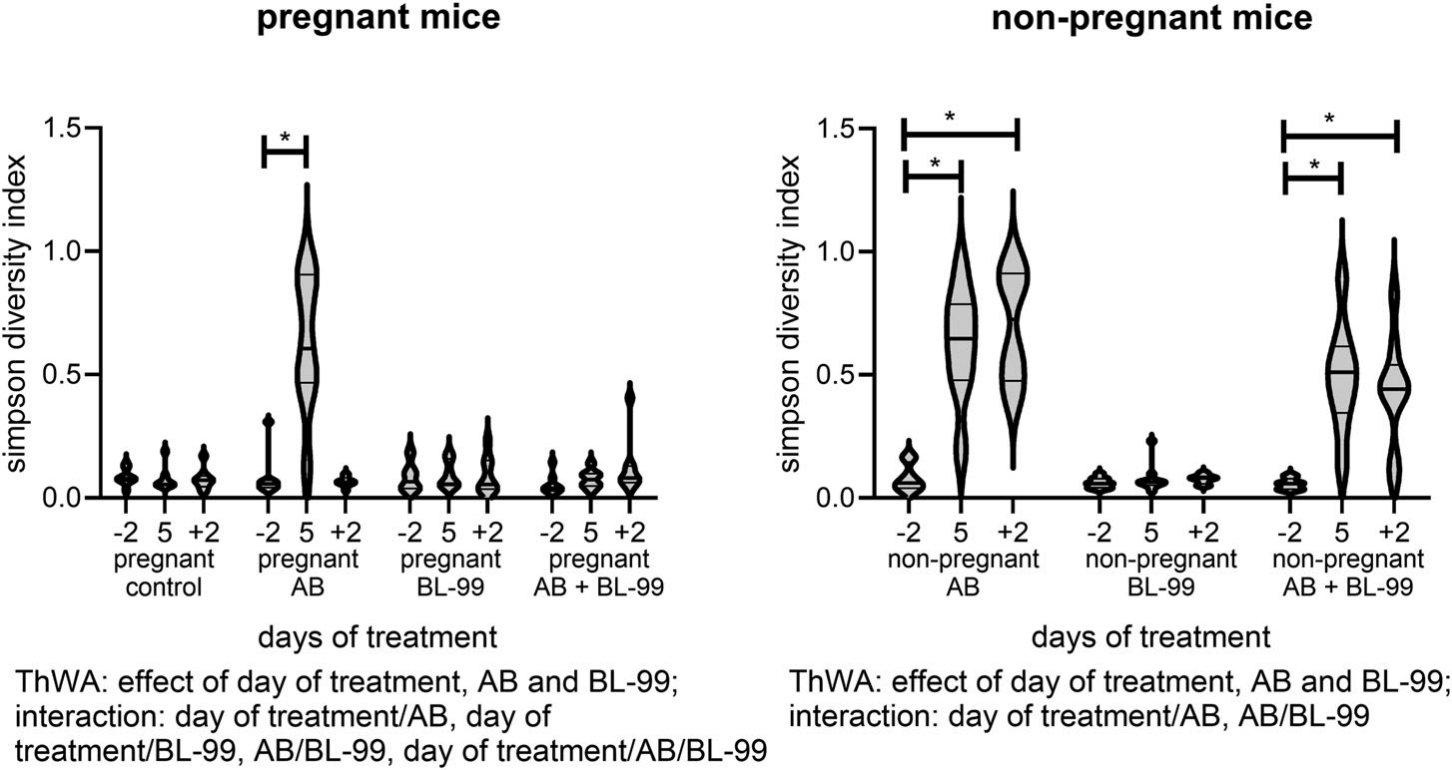
Simpson diversity indices of gut microbiota (genera) of pregnant (left) and nonpregnant (right) mice treated with or without antibiotic (AB) and supplemented with or without *Bifidobacterium animalis* subsp. *lactis* BL‐99 (BL‐99). Nonpregnant AB‐treated mice, pregnant AB‐treated mice, pregnant AB/BL‐99‐treated mice, pregnant control mice, and pregnant control mice supplemented with BL‐99 and nonpregnant control mice: 8 biological replicates; nonpregnant control mice supplemented with BL‐99 and nonpregnant AB/BL‐99‐treated mice: 9 biological replicates. For control nonpregnant mice, feces were only collected at Day −2 and not shown in the graph. *p < 0.05: Three‐way ANOVA (ThWA) followed by Sidak's multiple comparisons test.

Simpson diversity indices for pregnant and nonpregnant mice are shown in Figure 4. In pregnant mice (left graph), effects of AB, BL‐99, and day of treatment were found, while also all parameters interacted with each other (ThWA, *p* < 0.05). Posttests showed that the Simpson diversity index was increased after AB‐treatment on Day 5 as compared to Day −2 (Sidak's multiple comparison's test, *p* < 0.05). This returned to normal at Day +2 (Sidak's multiple comparison's test, *p* > 0.05 vs. Day −2). Treatment with BL‐99 abrogated the increasing effect of AB on Day 5 in pregnant mice (ThWA followed by Sidak's multiple comparison's test, *p* < 0.05). In nonpregnant mice (right graph), there was an effect of AB, as well as day of treatment, while there was an interaction between day of treatment and AB, and between AB and BL‐99 (ThWA, *p* < 0.05). Posttests in nonpregnant mice showed that AB treatment increased the Simpson diversity index at Days 5 and +2 (Sidak's multiple comparison's test, *p* < 0.05) versus Day −2, while BL‐99 supplementation did not abrogate this effect of AB treatment (ThWA, *p* > 0.05). BL‐99 treatment of control nonpregnant mice did not affect the Simpson diversity index (ThWA, *p* > 0.05).

**FIGURE 5:**
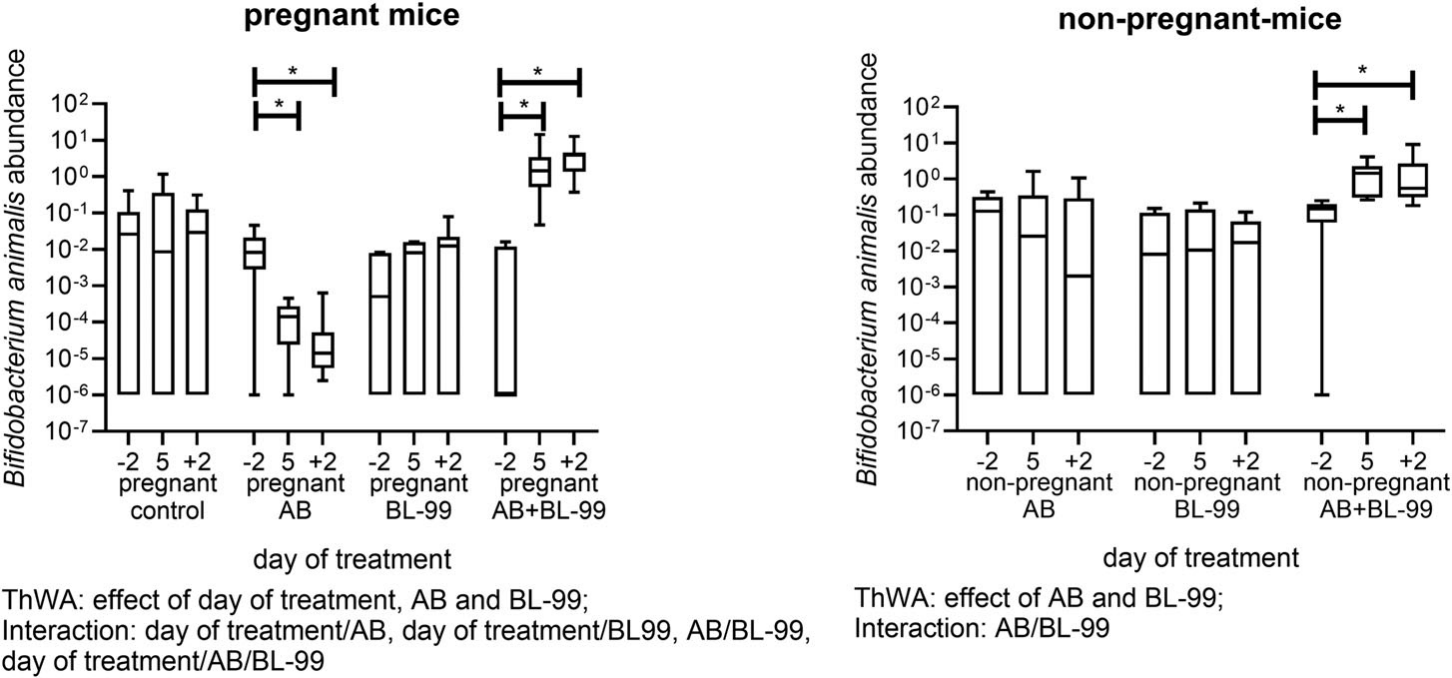
Abundance of *Bifidobacterium animalis* species in pregnant (left) and nonpregnant (right) mice treated with or without antibiotic (AB) and supplemented with or without *Bifidobacterium animalis* subsp. *lactis* BL‐99 (BL‐99). Nonpregnant AB‐treated mice, pregnant AB‐treated mice, pregnant AB/BL‐99‐treated mice, pregnant control mice, and pregnant control mice supplemented with BL‐99 and nonpregnant control mice: 8 biological replicates; nonpregnant control mice supplemented with BL‐99 and nonpregnant AB/BL‐99‐treated mice: 9 biological replicates. For control nonpregnant mice, feces were only collected at Day −2 and not shown in the graph. *p < 0.05: Three‐way ANOVA (ThWA) followed by Sidak's multiple comparisons test.

Using 16S rRNA data, we evaluated the abundance of *B. animalis* species (as a measure of the presence of BL‐99) in all fecal samples of pregnant and nonpregnant mice (Figure 5). *B. animalis* species were present in pregnant and nonpregnant untreated mice before the start of the treatment (Day −2). It can be observed that in pregnant mice, AB treatment significantly decreased the abundance of *B. animalis* species (ThWA followed by Sidak's multiple comparisons test, *p* < 0.05). Supplementation with BL‐99 increased the abundance of *B. animalis* species in pregnant AB‐treated mice (ThWA followed by Sidak's multiple comparisons test, *p* < 0.05). Also in nonpregnant mice, *B. animalis* species were present in all groups at Day −2. It can be observed that in nonpregnant mice, AB treatment did not affect the abundance of *B. animalis* species (ThWA followed by Sidak's multiple comparisons test, *p* > 0.05). Treatment with BL‐99 increased the abundance of *B. animalis* species in nonpregnant AB‐treated mice (ThWA followed by Sidak's multiple comparisons test, *p* < 0.05).

### 3.3 Effect of Treatment With AB and BL‐99 on the Intestinal Immune Response

**FIGURE 6:**
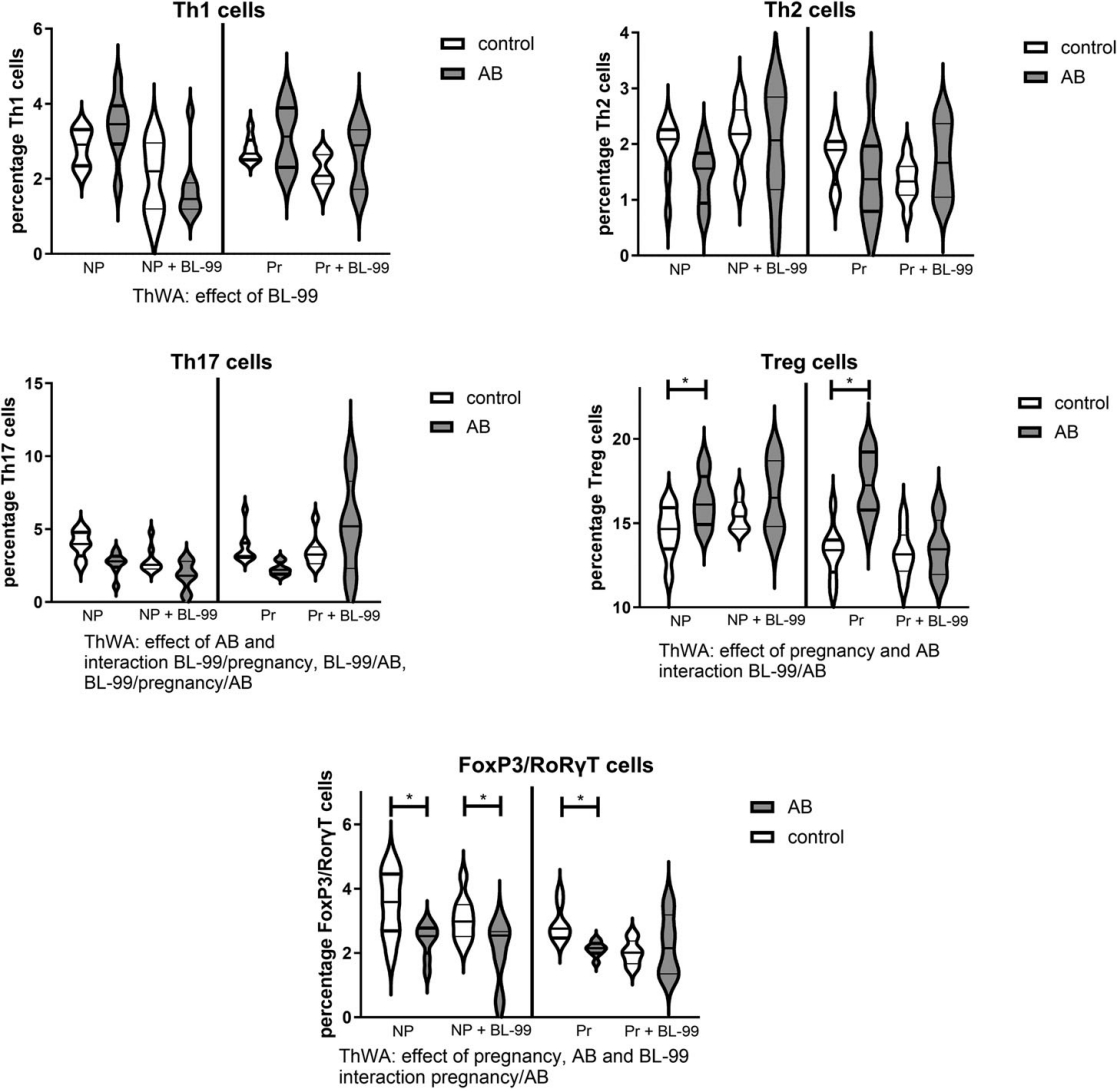
Percentage T helper (Th)1, Th2, Th17, Treg, and FoxP3/RoRγT double positive cells within the CD4 positive cell population in the Peyer's patches (PP) of pregnant and nonpregnant antibiotics (AB)‐treated mice with or without supplementation with *Bifidobacterium animalis* subsp. *lactis* BL‐99 (BL‐99). Nonpregnant AB‐treated mice, pregnant AB‐treated mice, pregnant AB/BL‐99‐treated mice, pregnant control mice, and pregnant control mice supplemented with BL‐99 and nonpregnant control mice: 8 biological replicates; nonpregnant control mice supplemented with BL‐99 and nonpregnant AB/BL‐99‐treated mice: 9 biological replicates. *p < 0.05: Three‐way ANOVA (ThWA) followed by Sidak's multiple comparisons test.

**FIGURE 7:**
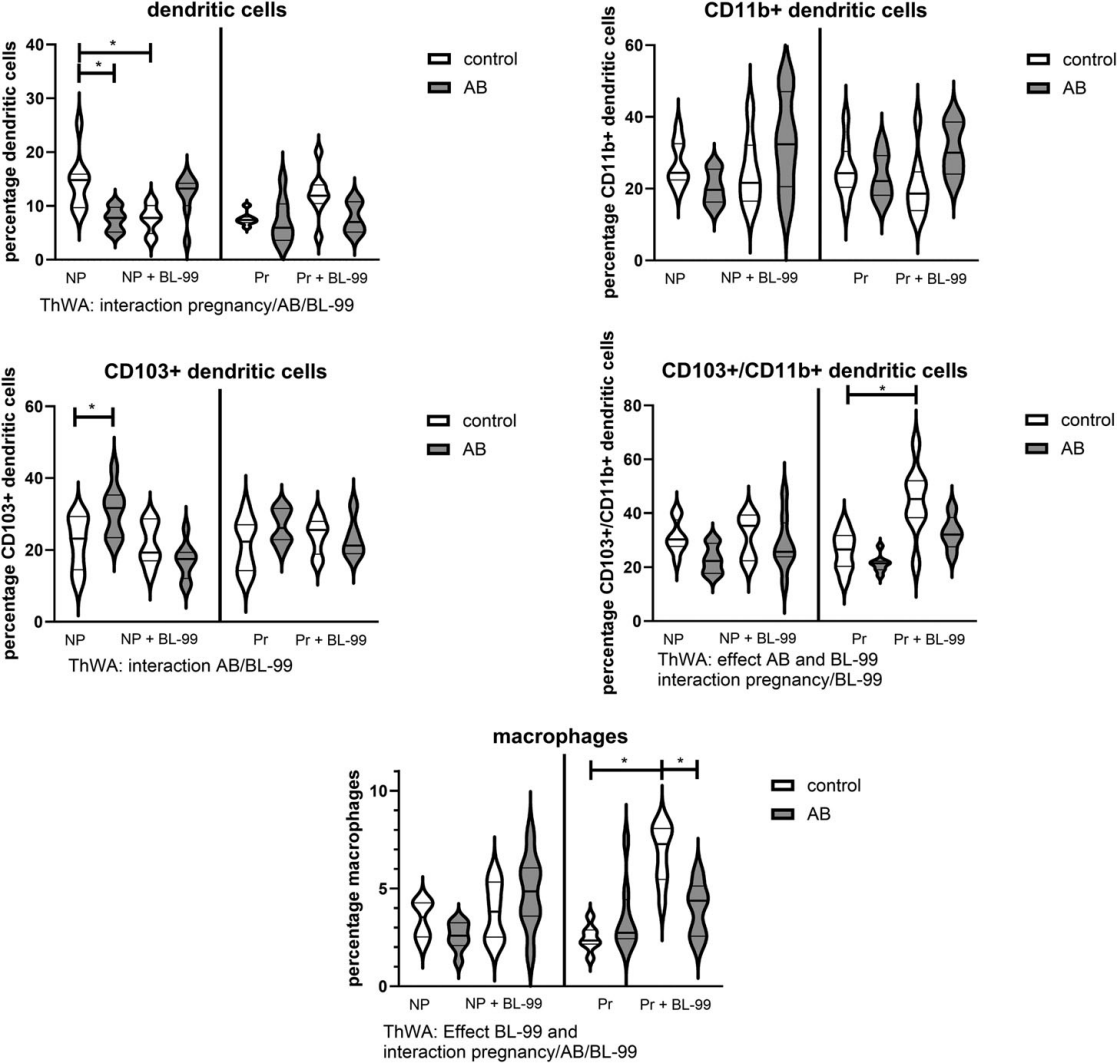
Dendritic cell subsets and macrophages in the Peyer's patches (PP) of pregnant and nonpregnant antibiotics (AB)‐treated and control mice supplemented with or without *Bifidobacterium animalis* subsp. *lactis* BL‐99 (BL‐99). Nonpregnant AB‐treated mice, pregnant AB‐treated mice, pregnant AB/BL‐99‐treated mice, pregnant control mice, and pregnant control mice supplemented with BL‐99 and nonpregnant control mice: 8 biological replicates; nonpregnant control mice supplemented with BL‐99 and nonpregnant AB/BL‐99‐treated mice: 9 biological replicates. *p < 0.05: Three‐way ANOVA (ThWA) followed by Sidak's multiple comparisons test.

We evaluated Th subsets (Figure 6) and dendritic cells (Figure 7) in the PP to evaluate the intestinal immune response. Th1 cells were affected, that is, slightly decreased, by BL‐99 in the PP of nonpregnant and pregnant mice (Figure 6, ThWA, *p* < 0.05). The Th2 cells were not affected by pregnancy, AB, or BL‐99 (ThWA, *p* > 0.05). Th17 cells in the PP were affected by AB, and there were interactions between AB treatment, BL‐99 supplementation, and treatment day. The graph shows that AB decreased Th17 cells in the PP; this effect was abolished by BL‐99 only in pregnant mice (ThWA, *p* < 0.05). In the posttest, these effects were not significant. Treg cells in the PP were affected by pregnancy, AB treatment, and there was an interaction between BL‐99 and AB. Posttesting showed that AB increased Treg cells in the PP of both nonpregnant and pregnant mice, while this effect was abolished by BL‐99 treatment (Sidak's multiple comparisons test, *p* < 0.05). FoxP3/RoRγT double‐positive cells in the PP were affected by pregnancy, AB, and BL‐99, and there was an interaction between pregnancy and AB (ThWA, *p* < 0.05). Posttesting indicated that AB significantly decreased FoxP3/ RoRγT double‐positive cells in both pregnant and nonpregnant mice, while this effect was only abolished by BL‐99 in pregnant mice, not in nonpregnant mice.

Figure 7 shows the percentages of dendritic cell populations and macrophages in the PPs. For dendritic cells (top left graph), there is an interaction between pregnancy, AB‐treatment, and BL‐99 supplementation (ThWA, *p* < 0.05). Posttesting showed that AB treatment decreased the percentage of dendritic cells in the PP of nonpregnant mice, while also BL‐99 supplementation decreased the percentage of dendritic cells in nonpregnant mice (Sidak's multiple comparisons test, *p* < 0.05). CD11b+ dendritic cells (top right graph) were not affected by pregnancy, AB treatment, or by BL‐99 supplementation (ThWA, *p* > 0.05). For CD103+ dendritic cells (middle left graph), we found an interaction between AB‐treatment and BL‐99 supplementation. Posttesting showed that CD103+ dendritic cells were increased by AB treatment in nonpregnant mice, an effect which was abrogated by BL‐99 supplementation (Sidak's multiple comparisons test, *p* < 0.05). For CD103+/CD11b+ dendritic cells (middle right graph), an effect was found for AB‐treatment and BL‐99 supplementation (ThWA, *p* < 0.05). Posttesting indicated that these cells were increased by BL‐99 supplementation in control pregnant mice (Sidak's multiple comparisons test, *p* < 0.05). The percentage of macrophages (lower graph) was affected by BL‐99 supplementation, and an interaction was found for pregnancy, AB‐treatment, and BL‐99 supplementation (ThWA, *p* < 0.05). We observed that macrophages were significantly increased by BL‐99 in pregnant mice (Sidak's multiple comparisons test, *p* < 0.05) and decreased by AB‐treatment in BL‐99‐supplemented pregnant mice (Sidak's multiple comparisons test, *p* < 0.05).

### 3.4 Effect of Treatment With AB and BL‐99 on the Peripheral Immune Response

**FIGURE 8:**
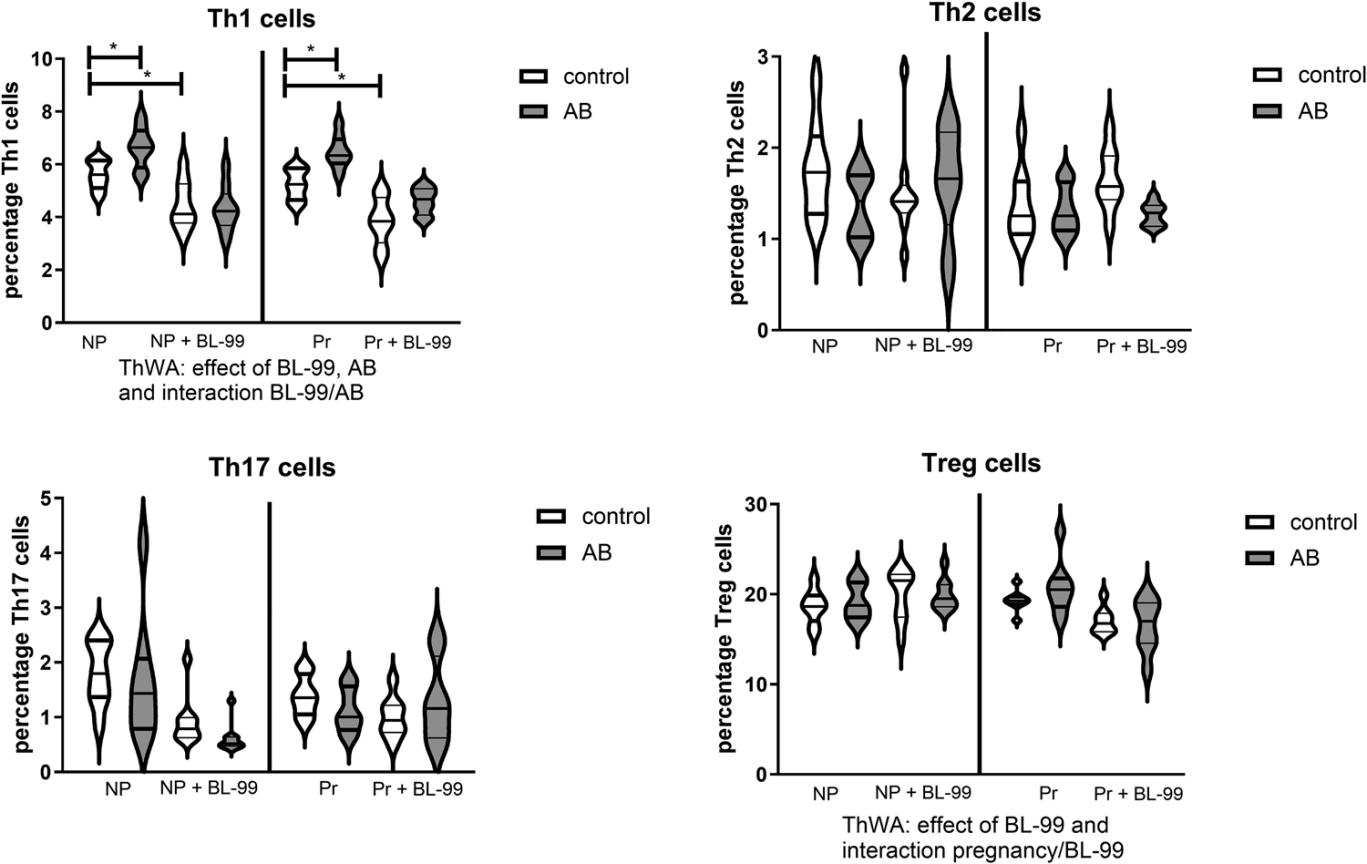
Percentage T helper (Th)1, Th2, Th17, and Treg cells within the CD4 positive cell population in the spleen of pregnant and nonpregnant antibiotics (AB)‐treated mice with or without supplementation with *Bifidobacterium animalis* subsp. *lactis* BL‐99 (BL‐99). Nonpregnant AB‐treated mice, pregnant AB‐treated mice, pregnant AB/BL‐99‐treated mice, pregnant control mice, and pregnant control mice supplemented with BL‐99 and nonpregnant control mice: 8 biological replicates; nonpregnant control mice supplemented with BL‐99 and nonpregnant AB/BL‐99‐treated mice: 9 biological replicates. *p < 0.05: Three‐way ANOVA (ThWA) followed by Sidak's multiple comparisons test.

Figure 8 shows the effect of AB treatment and BL‐99 supplementation on Th1, Th2, Th17, and Treg cells in the spleen of the various groups of mice. We observed an effect of AB and BL‐99 on Th1 cells in the spleen (ThWA, *p* < 0.05). Posttesting indicated that AB increased Th1 cells in both nonpregnant and pregnant mice. This effect of AB in nonpregnant and pregnant mice was abolished by BL‐99 treatment, since in AB/BL‐99‐treated mice, the Th1 cells were not different from pregnant or nonpregnant mice not treated with AB (Sidak's multiple comparisons test, *p* > 0.05). Moreover, BL‐99 supplementation per se decreased Th1 cells in control nonpregnant and pregnant mice (Sidak's multiple comparisons test, *p* < 0.05). Furthermore, we found an effect of BL‐99 on Treg cells and an interaction between BL‐99 and pregnancy (ThWA, *p* < 0.05). We found no effect of AB or BL‐99 on Th2 and Th17 cells in the spleen.

**FIGURE 9:**
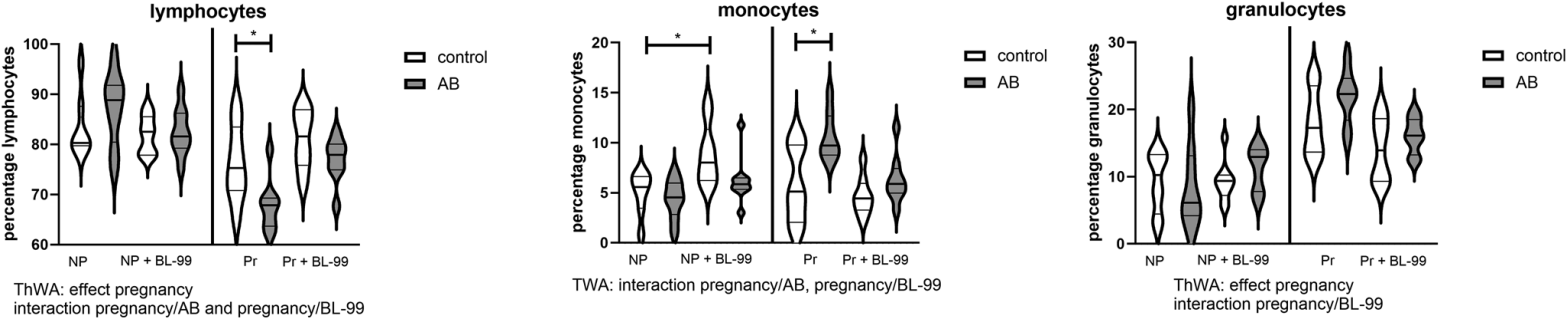
Effect of antibiotics (AB)‐treatment and *Bifidobacterium animalis* subsp. *lactis* BL‐99 (BL‐99) supplementation on circulating lymphocytes, monocytes, and granulocytes in pregnant and nonpregnant mice. Nonpregnant AB‐treated mice, pregnant AB‐treated mice, pregnant AB/BL‐99‐treated mice, pregnant control mice, and pregnant control mice supplemented with BL‐99 and nonpregnant control mice: 8 biological replicates; nonpregnant control mice supplemented with BL‐99 and nonpregnant AB/BL‐99‐treated mice: 9 biological replicates. *p < 0.05: Three‐way ANOVA (ThWA) followed by Sidak's multiple comparisons test.

The percentages of lymphocytes, monocytes, and granulocytes in the blood were also affected by AB and BL‐99 treatment (Figure 9). Lymphocyte numbers were affected by pregnancy, and there was an interaction between pregnancy and AB‐treatment and between pregnancy and BL‐99 supplementation (ThWA, *p* < 0.05). Posttesting showed that AB treatment significantly decreased lymphocyte numbers in pregnant mice; this effect of AB was abolished by BL‐99 supplementation (Sidak's multiple comparisons test, *p* < 0.05). For monocyte numbers, there was an interaction between pregnancy and AB‐treatment as well as between pregnancy and BL‐99 supplementation (ThWA, *p* < 0.05). Sidak's multiple comparisons test showed that in nonpregnant control mice, BL‐99 supplementation increased monocyte numbers. In pregnant mice, AB‐treatment decreased monocyte numbers (*p* < 0.05). This effect was inhibited by BL‐99 supplementation. Granulocyte numbers were affected by pregnancy, and there was an interaction between pregnancy and BL‐99 supplementation (ThWA, *p* < 0.05).

**FIGURE 10:**
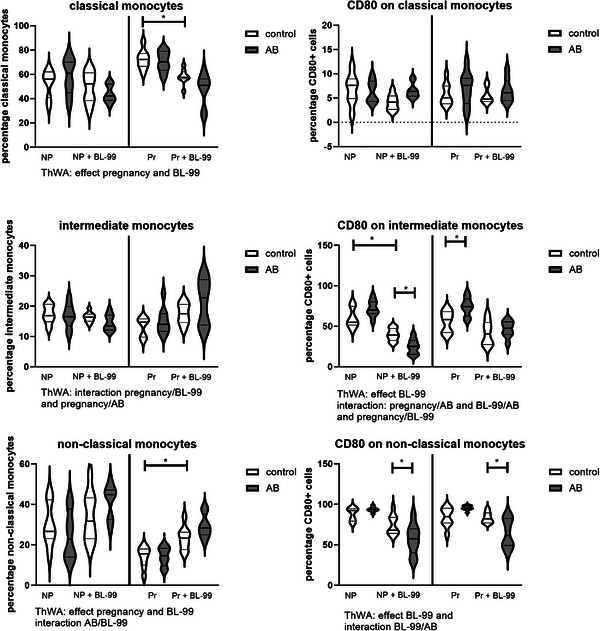
Monocyte subsets and their CD80 expression in pregnant and nonpregnant mice treated with or without antibiotics (AB) and supplemented with or without *Bifidobacterium animalis* subsp. *lactis* BL‐99 (BL‐99). Nonpregnant AB‐treated mice, pregnant AB‐treated mice, pregnant AB/BL‐99‐treated mice, pregnant control mice, and pregnant control mice supplemented with BL‐99 and nonpregnant control mice: 8 biological replicates; nonpregnant control mice supplemented with BL‐99 and nonpregnant AB/BL‐99‐treated mice: 9 biological replicates. *p < 0.05: Three‐way ANOVA (ThWA) followed by Sidak's multiple comparisons test.

Monocyte subsets and their activation status as measured by CD80 expression (Figure 10) were also affected by AB treatment and BL‐99 supplementation. In classical monocytes, we observed an effect of pregnancy and BL‐99 supplementation. Posttesting showed that BL‐99 supplementation decreased classical monocytes in pregnant mice. In intermediate monocytes, we found an interaction between pregnancy and BL‐99 supplementation and between pregnancy and AB‐treatment. Posttesting did not show significant differences between the individual groups. In nonclassical monocytes, we found an effect of pregnancy and BL‐99 supplementation and an interaction between BL‐99 supplementation and AB‐treatment (ThWA, *p* < 0.05). Posttesting showed that BL‐99 supplementation increased nonclassical monocytes in pregnant mice (Sidak's multiple comparisons test, *p* < 0.05). Prominent effects were also shown on monocyte CD80 expression (Figure 10). The percentage of classical monocytes expressing CD80 was not affected by either pregnancy, AB treatment, or BL‐99 supplementation (ThWA, *p* > 0.05). The percentage of intermediate monocytes expressing CD80 was affected by BL‐99 supplementation, while an interaction between pregnancy and BL‐99 supplementation, between pregnancy and AB‐treatment, and between AB‐treatment and BL‐99 supplementation was observed (ThWA, *p* < 0.05). Posttesting showed that BL‐99 supplementation decreased the percentage of CD80‐expressing intermediate monocytes in nonpregnant control mice and AB‐treated mice (Sidak's multiple comparisons test, *p* < 0.05). In pregnant mice, AB‐treatment increased the percentage of CD80‐expressing intermediate monocytes. This AB‐effect was abolished by BL‐99 supplementation in these mice (Sidak's multiple comparisons test, *p* < 0.05). The percentage of nonclassical monocytes expressing CD80 was affected by BL‐99 supplementation, and an interaction between BL‐99 supplementation and AB‐treatment was observed (ThWA, *p* < 0.05). Posttesting indicated that in both pregnant and nonpregnant mice, BL‐99 supplementation decreased the percentage of CD80‐expressing nonclassical monocytes in AB‐treated mice (Sidak's multiple comparisons test, *p* < 0.05).

### 3.5 Effect of Treatment With AB and BL‐99 on the Fetal Immune Response

**FIGURE 11:**
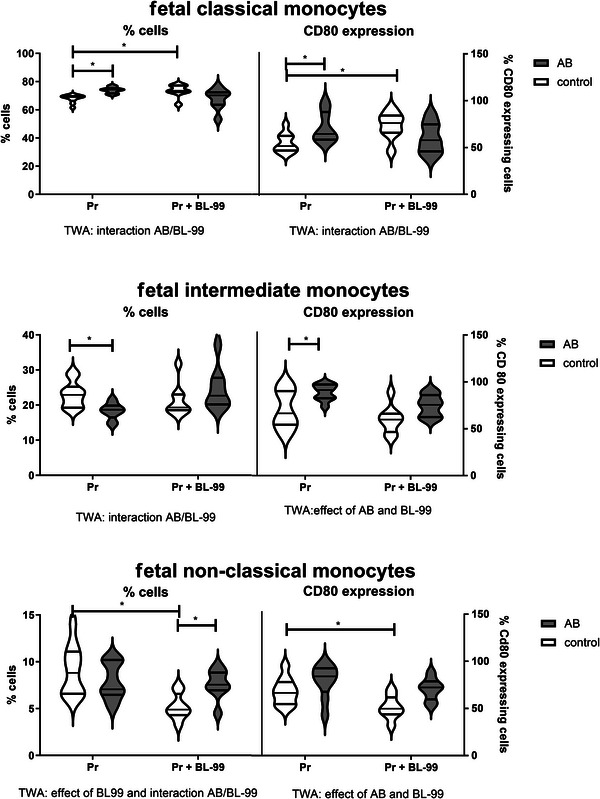
Fetal monocytes in pregnant mice treated with or without antibiotics (AB) and supplemented with or without *Bifidobacterium animalis* subsp. *lactis* BL‐99 (BL‐99). Pregnant AB‐treated mice, pregnant AB/BL‐99‐treated mice, pregnant control mice, and pregnant control mice supplemented with BL‐99: 8 biological replicates. *p < 0.05: Two‐way ANOVA (TWA) followed by Sidak's multiple comparisons test.

Not only maternal immune cells but also fetal immune cells were affected by AB treatment and BL‐99 supplementation. Figure 11 shows the changes in fetal monocyte subsets and their CD80 expression. Maternal AB‐treatment affected fetal monocyte subpopulations; it increased the percentage of fetal classical monocytes, while it decreased the percentage of fetal intermediate monocytes as compared to control untreated pregnant mice (TWA followed by Sidak's multiple comparisons test, *p* < 0.05). Maternal BL‐99 supplementation increased the percentage of fetal classical monocytes, while it decreased the percentage of fetal nonclassical monocytes (TWA followed by Sidak's multiple comparisons test, *p* < 0.05). Furthermore, AB treatment affected the activational status of fetal monocytes. It increased the expression of CD80 on classical and intermediate fetal monocytes as compared with untreated pregnant mice (TWA followed by Sidak's multiple comparisons test, *p* < 0.05). Supplementation with BL‐99 increased CD80 expression on classical monocytes and decreased CD80 expression on nonclassical fetal monocytes as compared to untreated pregnant mice (TWA followed by Sidak's multiple comparisons test, *p* < 0.05).

The TWA indicated an effect of BL‐99 supplementation on the percentage of Th cells in the fetal spleen (Figure 5). The fetal spleen of pregnant untreated and AB‐treated mice hardly contained Th cells; however, supplementation with BL‐99 increased the percentage of Th cells in the spleens in both control and AB‐treated mice (TWA, *p* < 0.05). In view of the small number of Th cells in the fetal spleen, it was not possible to reliably analyze Th cell subsets from the fetal spleen.

### 3.6 Correlation Between *Bifidobacterium animalis* in the Maternal Feces and Maternal/Fetal Immune Cells

**FIGURE 12:**
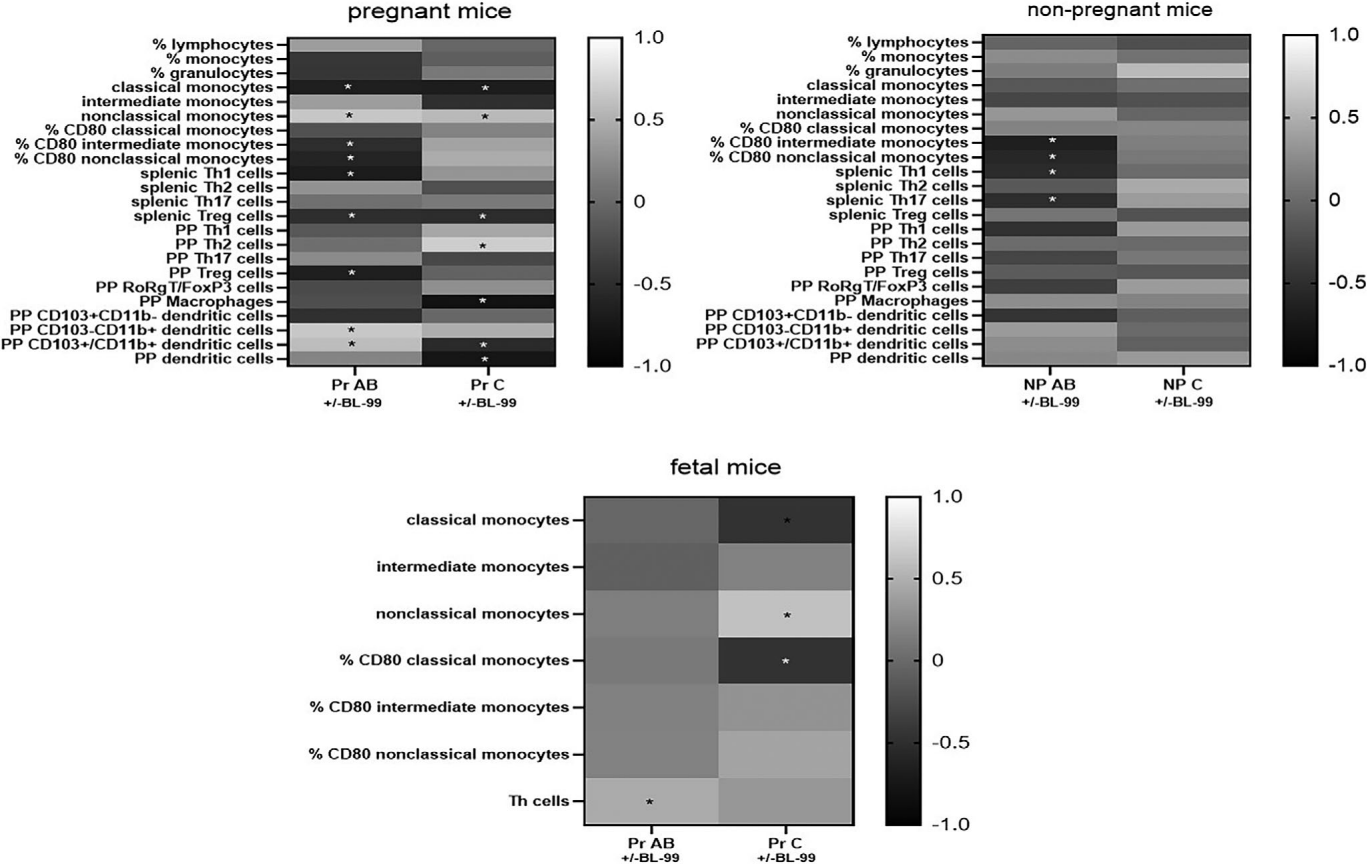
Correlation of immune cell subsets of pregnant (maternal (top left) and fetal (bottom) and nonpregnant mice (top right) with *Bifidobacterium animalis* abundance in the feces. NTheonpregnant antibiotic (AB)‐treated mice, pregnant AB‐treated mice, pregnant AB/*Bifidobacterium animalis* subsp. *lactis* BL‐99 (BL‐99)‐treated mice, pregnant control mice, and pregnant control mice supplemented with BL‐99 and nonpregnant control mice: 8 biological replicates; nonpregnant control mice supplemented with BL‐99 and nonpregnant AB/BL‐99‐treated mice: 9 biological replicates.

To further evaluate the effect of BL‐99 on AB‐treated mice, we correlated the abundance of *B. animalis* (as a measure for the abundance of BL‐99) with immune cells and their activation markers of AB‐treated and control pregnant and nonpregnant mice (Figure 12). We found that various immune cell populations correlated significantly with the abundance of *B. animalis* in pregnant AB‐treated mice, such as the classical and nonclassical monocytes and CD80 expression on intermediate and nonclassical monocytes, Th1 cells in the spleen, Treg cells in the spleens, and PP, as well as with subsets of dendritic cells in the PP. Also in pregnant control mice, that is, mice not treated with AB, the abundance of *B. animalis* was correlated with various immune cells, such as monocytes subsets, regulatory T cells in the spleen, and dendritic cells in the PP. Correlations between *B. animalis* and immune cells in nonpregnant mice are also observed, but only with cells in the spleen and blood not with cells in the PP. Also, fetal immune cells are correlated with the presence of *B. animalis* in the maternal feces. In AB‐treated mice, the percentage of splenic Th cells correlated positively with *B. animalis* in the maternal feces. In control pregnant mice, fetal classical monocytes and the percentage of CD80‐expressing fetal classical monocytes correlated negatively with the abundance of *B. animalis*, while fetal nonclassical monocytes correlated positively with the abundance of *B. animalis* species.

## 4 Discussion

Treatment of pregnant mice with AB disturbed the maternal gut microbiota, affected maternal immune responses, and decreased fetal and placental weight [16]. Additionally, we now show that maternal AB‐treatment also affected fetal immune cells, especially monocytes. Moreover, we showed that simultaneous supplementation with BL‐99 increased the abundance of *B. animalis* species in the maternal gut microbiota, which resulted in improved maternal immune responses and increased placental weight, and an improved fetal immune response. Since our study showed that the numbers of various maternal and fetal immune cells are correlated with the abundance of *B. animalis* species, which reflects the increase in BL‐99, we suggest that the increase in BL‐99 in the maternal gut improved maternal immune responses, resulting in increased placental weight.

BL‐99 supplementation increased placental weight, but not fetal weight, in both AB‐treated and control pregnant mice, suggesting a role for BL‐99 in placental growth. This suggests that in this model BL‐99 supplementation increased placental growth, but did not improve placental function. Lopez‐Tello et al. also evaluated the effect of *Bifidobacteria* on pregnancy [26]. They, however, found an increase in fetal weight, but not in placental weight. Differences could be due to the use of a different animal model, that is, germfree mice, and a different Bifidobacterium species, that is, *Bifidobacterium breve*. Since *Bifidobacteria* are known for their effects on immune responses [27, 28], we hypothesize that AB‐treatment deranged placental immune cells, which are important for placental growth and development [29], causing decreased placental growth. This hypothesis is in line with the results of a previous study from Benner et al. [25], showing an effect of maternal dysbiosis on placental immune responses. We are currently evaluating placental immune responses after AB and BL‐99 treatment.

BL‐99 supplementation in control pregnant mice increased CD103/CD11b double‐positive cells in the PP, decreased Th1 cells in the spleen, and decreased classical monocytes and increased nonclassical monocytes in the blood. *Bifidobacteria* are well‐known for their effects on immune responses, especially in neonates [27, 28]. They have immunomodulatory and antiinflammatory properties by upregulating Treg cells [30], increasing Th1 and decreasing Th2 and Th17 immune responses [30, 31]. We found a different effect of BL‐99, which was a decrease in the percentage of Th1 cells and no effect on Treg cells in the spleens of pregnant mice. Our results, however, are in line with other experimental animal studies: treatment of rats or mice with experimental autoimmune encephalomyelitis with *Bifidobacteria* in combination with *Lactobacillus* or another probiotic mixture decreased peripheral Th1 and increased peripheral Th2 responses [32, 33]. Potentially, *Bifidobacteria* have multipotent immunomodulatory effects, depending on species or strains [34], but also depending on the use in adults or neonates or in pregnant versus nonpregnant mice.

Supplementation of AB‐treated pregnant mice with BL‐99 mitigated many of the AB‐induced immune aberrations in pregnant mice. The increased splenic Th1 cells, increased percentage Treg cells, and decreased percentage of FoxP3/RoRγT double‐positive cells in the PP after AB‐treatment were not observed in AB‐treated pregnant mice supplemented with BL‐99. For Treg cells and FoxP3/RoRγT double‐positive cells in the PP, this effect was only observed in pregnant mice, suggesting a pregnancy‐specific effect of BL‐99 on the T cells in the PP. This pregnancy‐specific effect is confirmed by our correlation studies, which only showed a significant correlation between immune cells in the PP and the presence of BL‐99 in pregnant mice. Whether this pregnancy‐specific effect of BL‐99 on immune cells in the PP is due to the changes in the microbiome during pregnancy, and how this immune effect in the PP is translated to better pregnancy outcome remains to be established.

The present study showed that not only the maternal immune system but also the fetal immune system is affected by both AB‐treatment and BL‐99 supplementation. Maternal AB treatment increased the percentage of fetal classical monocytes and decreased the percentage of fetal intermediate monocytes, while it also increased CD80 expression on fetal classical and intermediate monocytes. These effects were mitigated by BL‐99 treatment. Since we used nonabsorbable AB [16, 20], this effect is not induced by a direct effect of AB on the fetuses. Recent insights into the development of the fetal immune response have shown a role of the maternal gut microbiome in the development of the fetal immune responses [35]. Therefore, it seems likely that in the present study, the changes in the maternal microbiome and the increased presence of BL‐99 affected the fetal immune cells. The maternal microbiome and BL‐99 could affect the fetal immune response by transfer of bacterial products, such as bacterial DNA or short‐chain fatty acids, across the placenta [35, 36]. Whether these changes in the fetal immune response have consequences for long‐term effects on the offspring's immune response is difficult to predict from the present study. However, it is known that prenatal maternal AB treatment increases the risk of asthma in childhood, suggesting long‐term effects of maternal AB treatment on the fetal immune response [37]. Future studies should thus evaluate whether the observed changes induced by AB or BL‐99 treatment result in long‐term changes in the offspring's immune response and an increased risk for the development of immune‐mediated diseases, such as autoimmune diseases or asthma.

Various differences in the response to AB treatment or BL‐99 supplementation between pregnant and nonpregnant mice were observed. For instance, maternal monocyte subsets are only affected by BL‐99 supplementation in pregnant mice, while AB treatment only affected dendritic cells and CD103+ dendritic cells in the PP of nonpregnant mice. Also, the impact of AB‐treatment on the gut microbiome differs between pregnant and nonpregnant mice. The reason for these differences is unknown but may lie in the differences in microbiome composition between pregnant and nonpregnant mice [14]. Alternatively, it may result from differences in the sensitivity of immune cells between pregnant and nonpregnant mice: immune cells from pregnant and nonpregnant mice may respond differently to gut microbial changes. Future studies should discriminate between these options.

## 5 Concluding Remarks

In the present study, we showed that disturbance in the maternal microbiome by AB affected maternal and fetal immune cells. This effect was largely mitigated by simultaneous supplementation with the probiotic BL‐99. As one possible mechanism for this protective effect of BL‐99, we investigated the effects of BL‐99 treatment on the immune response of pregnant and nonpregnant AB‐treated mice. The decreasing effect of BL‐99 on the peripheral Th1 response may be (partly) responsible for the improved pregnancy outcome, since increased Th1 responses are associated with various pregnancy complications, such as preeclampsia and fetal growth restriction [8]. Also the effects of BL‐99 on monocytes, especially the decreased activational status in AB‐treated pregnant mice after BL‐99 supplementation, may be related to improved fetal outcome, since monocyte activation is also associated with complications as preeclampsia and fetal growth retardation [4]. Future studies should further evaluate the mechanism by which BL‐99 induces its effect, including measurement of absolute numbers of immune cells, functional measurements of the immune cells, and measurement of the gut metabolome as well as circulating short‐chain fatty acids and cytokines.

Our study may have relevance for human studies. Pregnant and nonpregnant mice respond differently to BL‐99. This may suggest that results from studies with probiotics in nonpregnant (or male) individuals cannot be directly translated to pregnant women, and that clinical studies with probiotics should be performed in pregnant individuals before the use of probiotics during pregnancy can be promoted. There are various indications that, like in the present study, also in humans AB‐treatment during pregnancy may affect the fetal and maternal immune response. It has for instance been shown that AB‐treatment during pregnancy is linked to increased risk of infection [38] or asthma [39] in the children, while the risk of preeclampsia, a pregnancy complication associated with a disturbed maternal immune response, may be increased in women who have used AB during pregnancy [40]. As our data show that the effects of AB treatment, that is, the effects of a disturbed microbiome, on the immune response of pregnant mice and their fetuses could largely be mitigated by BL‐99 treatment, future studies should focus on supplementing pregnant women with a disturbed microbiome and disturbed maternal immune responses with BL‐99.

## Conflicts of Interest

Carolien A. van Loo‐Bouwman, Gisela A. Weiss, and Wei‐lian Hung are employed by the company Yili Innovation Center Europe B.V., Wageningen, the Netherlands. The remaining authors declare that the research was conducted in the absence of any commercial or financial relationships that could be construed as a potential conflict of interest.

## Supporting information




**Supporting File 1**: mnfr70220‐sup‐0001‐SuppMat.docx.

## Data Availability

The data that support the findings of this study are available from the corresponding author upon reasonable request.
