# Supplementary material for: Bifidobacterium animalis subsp. Lactis BL‐99 Improves Maternal and Fetal Immune Responses and Pregnancy Outcomes in Pregnant Antibiotics‐Treated Mice
Source: Mol Nutr Food Res. 2025 Sep 4;69(21):e70220. doi: 10.1002/mnfr.70220 (PMC12581728; doi:10.1002/mnfr.70220)
Supplement: Supplementary file 1 — Supporting File 1: mnfr70220‐sup‐0001‐SuppMat.docx. [file MNFR-69-e70220-s001.docx]

**Supplementary files:**

Supplementary table 1: Antibodies used for staining of Th cell subsets, dendritic cells and monocytes.

**Th cells**

| **Marker** | **Fluorochrome** | **Dilution** | **Supplier** | **mix** |
| --- | --- | --- | --- | --- |
| CD4 | PerCp-Cy5.5 | 75x | Biolegend | Extracellular |
| CD3 | BV605 | 25x | Biolegend | Extracellular |
| Tbet | BV421 | 10x | Biolegend | Intracellular |
| RORγT | PE | 100x | eBioscience | Intracellular |
| Gata3 | AF647 | 100x | BD Pharmingen | Intracellular |
| FoxP3 | Fitc | 50x | eBioscience | intracellular |
| Dead/Live | Zombie NIR | 1000x | Biolegend |  |

**Dendritic cells**

| MHCII | PerCp-Cy5.5 | 1:200 | Biolegend |
| --- | --- | --- | --- |
| CD11c | APC | 1:50 | BD Pharmingen |
| CD64 | PE-Cy7 | 1:25 | Biolegend |
| CD19 | FITC | 1:25 | Biolegend |
| B220 | FITC | 1:25 | Biolegend |
| Nkp46 | FITC | 1:25 | Biolegend |
| CD103 | BV421 | 1:20 | Biolegend |
| CD11b | PE | 1:50 | Biolegend |
| Dead/live | Zombie green | 1:1000 | Biolegend |
|  |  |  |  |

**Monocytes**

| **Marker** | **Fluorochrome** | **Dilution** | **Supplier** |
| --- | --- | --- | --- |
| Ly6C | AF488 | 200x | Biolegend |
| CD43 | APC | 100x | Biolegend |
| CD11b | PE | 50x | Biolegend |
| CD80 | PB | 25x | Biolegend |
| Ly6G | BV605 | 25x | BD Horizon |
|  |  |  |  |


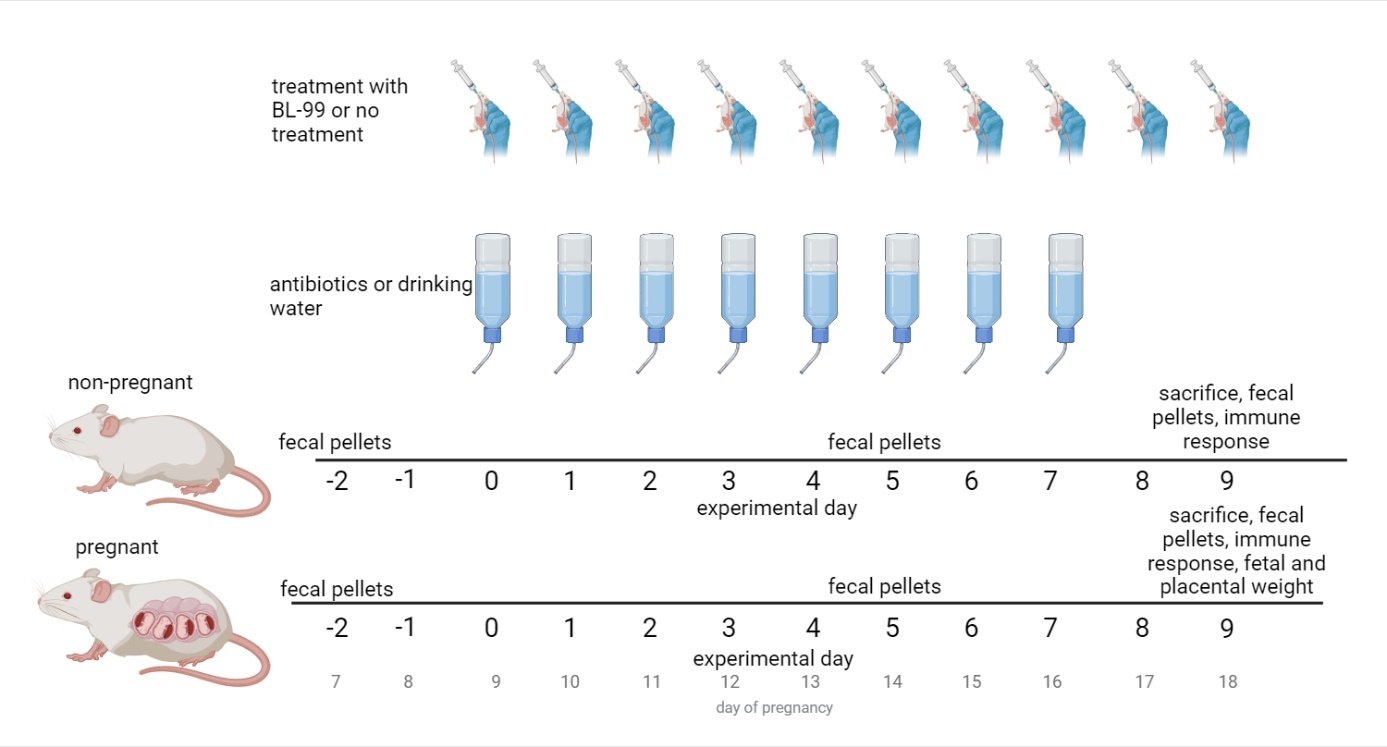


*Supplementary figure 1: Schematic representation of the experimental setup. All groups contained 8-9 biological replicates: pregnant AB-treated mice: n=8 (4 of these mice were randomly taken from our previous paper (Faas et al., 2023) and 4 mice are new); pregnant AB/BL-99 treated mice: n=8 (these mice are new); pregnant control mice: n=8 (4 of these mice were randomly taken from our previous paper (Faas et al., 2023) and 4 mice are new); pregnant control mice supplemented with BL-99: n=8 (these mice are new); non-pregnant AB-treated mice: n=8 (3 of these mice were randomly taken from our previous paper (Faas et al., 2023) and 5 mice are new); non-pregnant AB/BL-99 treated mice: n=9 (these mice are new); non-pregnant control mice: n=8 (these mice are new); non-pregnant control mice supplemented with BL-99: n=9 (these mice are new)*

*Supplementary figure 2: Weight changes of pregnant (left graph) and non-pregnant (right graph) mice treated or not treated with antibiotics (AB) and supplemented with or without BL-99. For pregnant mice: day 0 (start of the experiment) was day 9 of pregnancy. Day 9 of the experiment was day 18 of pregnancy, i.e. day of sacrifice. Non-pregnant AB-treated mice, pregnant AB-treated mice, pregnant AB/BL-99 treated mice, pregnant control mice, and pregnant control mice supplemented with BL-99 and non-pregnant control mice: 8 biological replicates; non-pregnant control mice supplemented with BL-99 and non-pregnant AB/BL-99 treated mice: 9 biological replicates.*

**antibiotics treated mice vs control mice on the same day: Mann-Whitney U test, p<0.05.*

*#: antibiotics/BL-99 treated mice vs antibiotics treated mice: Mann-Whitney U test, p<0.05.*


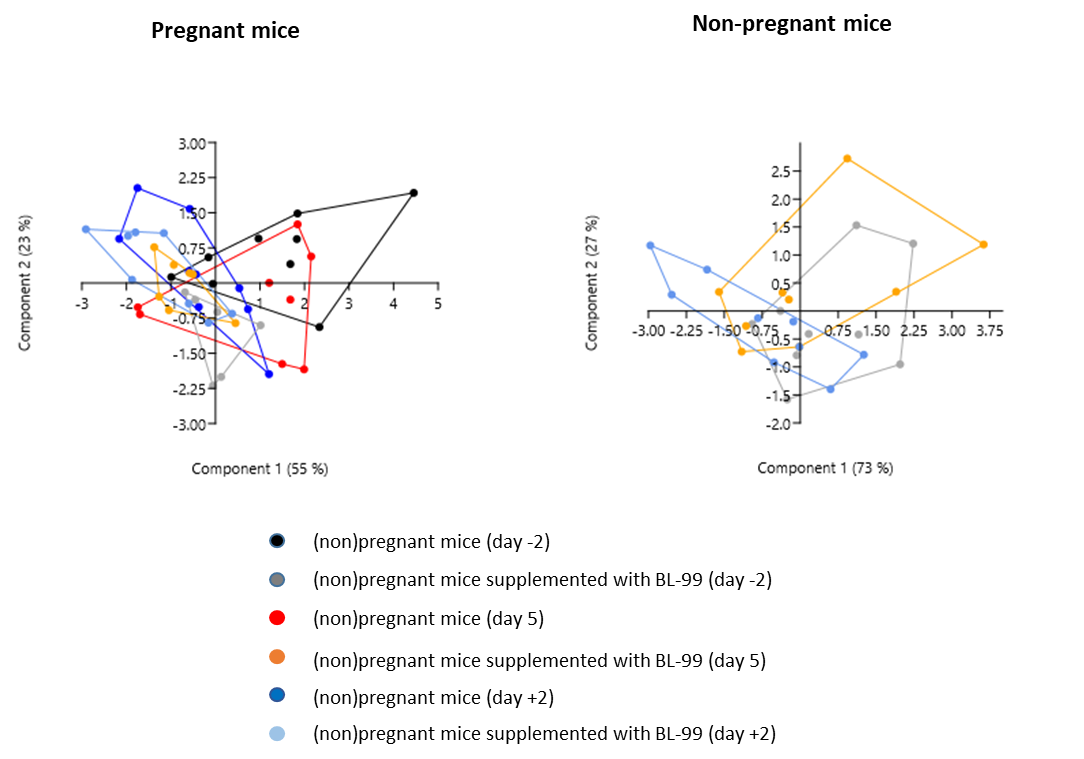


*Supplementary figure 3: PCA plots of bacterial phyla in pregnant (left) and non-pregnant (right) control mice only supplemented with or without BL-99.*

*Pregnant control mice, pregnant control mice supplemented with BL-99 and non-pregnant control mice: 8 biological replicates; non-pregnant control mice supplemented with BL-99: 9 biological replicates.*


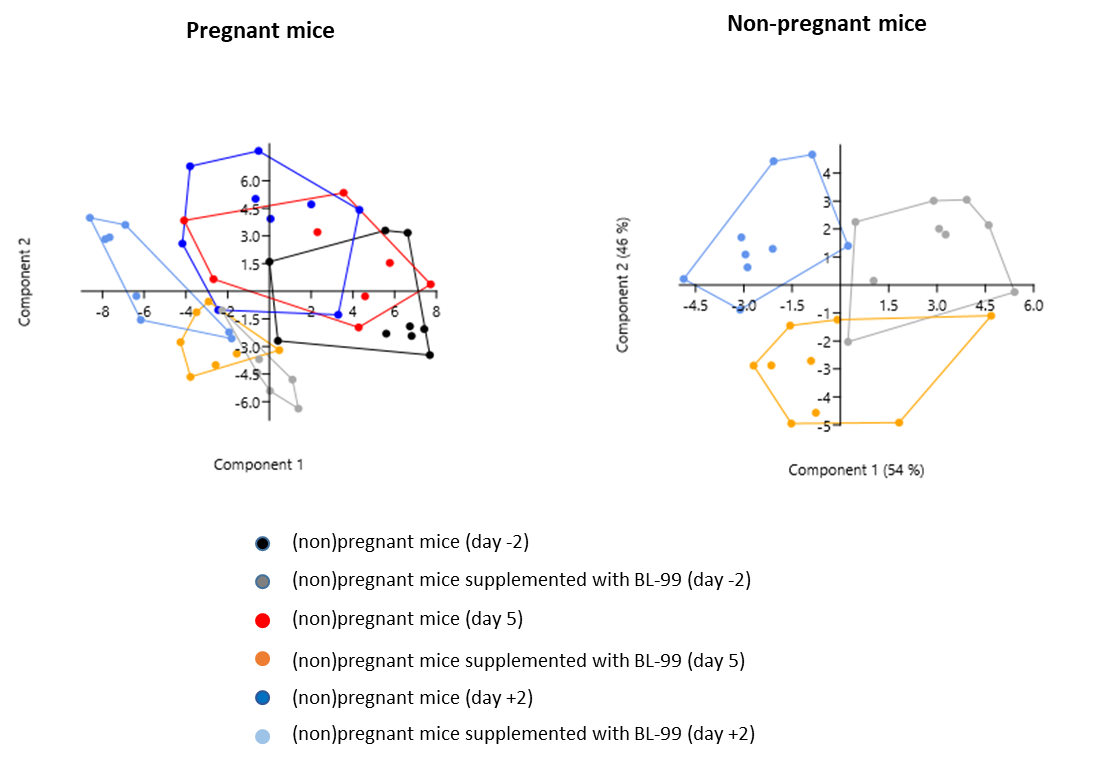


*Supplementary figure 4: PCA plots of bacterial genera in pregnant (left) and non-pregnant (right) control mice only supplemented with or without BL-99.*

*Pregnant control mice, pregnant control mice supplemented with BL-99 and non-pregnant control mice: 8 biological replicates; non-pregnant control mice supplemented with BL-99: 9 biological replicates.*

*Supplementary figure 5: Th cells in the fetal spleens of pregnant mice treated with or without antibiotics (AB) and supplemented with or without BL-99*. *Each group contains 8 biological replicates.*

*Two-way ANOVA (TWA) followed by Sidak’s Multiple comparisons test.*
